# Key Risk Genes Identified From the Postmortem Brain of Patients With Major Depressive Disorder and Their Potential Clinical Applications

**DOI:** 10.1093/ijnp/pyad024

**Published:** 2023-05-26

**Authors:** Qishuai Zhuang, Jingxian Wang, Xiaobing Li, Xiaoning Zhang, Yue Wang

**Affiliations:** Department of Neurosurgery, The First Affiliated Hospital of Shandong First Medical University and Shandong Provincial Qianfoshan Hospital, Jinan, China; Medical Science and Technology Innovation Center, Shandong First Medical University and Shandong Academy of Medical Sciences, Jinan, China; Department of Neurosurgery, The First Affiliated Hospital of Shandong First Medical University and Shandong Provincial Qianfoshan Hospital, Jinan, China; Medical Science and Technology Innovation Center, Shandong First Medical University and Shandong Academy of Medical Sciences, Jinan, China; Department of Neurosurgery, The First Affiliated Hospital of Shandong First Medical University and Shandong Provincial Qianfoshan Hospital, Jinan, China; Department of Neurosurgery, The First Affiliated Hospital of Shandong First Medical University and Shandong Provincial Qianfoshan Hospital, Jinan, China; Department of Neurosurgery, The First Affiliated Hospital of Shandong First Medical University and Shandong Provincial Qianfoshan Hospital, Jinan, China

**Keywords:** Differentially expressed genes, human brain, major depressive disorder, chronic unpredicted moderate stress, traditional Chinese medicine screening

## Abstract

**Background:**

Major depressive disorder (MDD) is a type of emotional dysfunction, and its pathogenesis has not been fully elucidated. Specifically, the key molecules in depression-related brain regions involved in this disease and their contributions to this disease are currently unclear.

**Methods:**

GSE53987 and GSE54568 were selected from the Gene Expression Omnibus database. The data were standardized to identify the common differentially expressed genes (DEGs) in the cortex of MDD patients in the 2 datasets. The DEGs were subjected to Gene Ontology and Kyoto Encyclopedia of Genes and Genomes pathway analyses. The STRING database was used to build protein–protein interaction networks, and the cytoHubba plugin was used to identify hub genes. Furthermore, we selected another blood transcriptome dataset that included 161 MDD and 169 control samples to explore the changes in the screened hub genes. Mice were subjected to 4 weeks of chronic unpredictable mild stress to establish an animal model of depression, and the expression of these hub genes in tissues of the prefrontal cortex was then detected by quantitative real time polymerase chain reaction (qRT-PCR). We subsequently predicted the possible posttranscriptional regulatory networks and traditional Chinese medicine according to the hub genes using a few online databases.

**Results:**

The analysis identified 147 upregulated genes and 402 downregulated genes were identified in the cortex of MDD patients compared with that of the controls. Enrichment analyses revealed that DEGs were predominantly enriched in synapse-related cell functions, linoleic acid metabolism, and other pathways. Protein–protein interaction analysis identified 20 hub genes based on the total score. The changes in *KDM6B*, *CUX2*, *NAAA*, *PHKB*, *NFYA*, *GTF2H1*, *CRK*, *CCNG2*, *ACER3*, and *SLC4A2* in the peripheral blood of MDD patients were consistent with those in the brain. Furthermore, the prefrontal cortex of mice with depressive-like behaviors showed significantly increased *Kdm6b*, *Aridb1*, *Scaf11*, and *Thoc2* expression and decreased *Ccng2* expression compared with that of normal mice, which was consistent with the results found for the human brain. Potential therapeutic candidates, such as citron, fructus citri, leaves of *Panax Notoginseng*, sanchi flower, pseudoginseng, and dan-shen root, were selected via traditional Chinese medicine screening.

**Conclusions:**

This study identified several novel hub genes in specific brain regions involved in the pathogenesis of MDD, which may not only deepen our understanding of depression but may also provide new ideas for its diagnosis and treatment.

**Significance Statement**

Major depressive disorder (MDD) is an emotional disorder caused by environmental and genetic factors. Determining the pathogenic genes involved in the occurrence and development of MDD, especially in some specific depression-related brain regions, is very important and valuable, but relatively few studies have investigated these genes, and most of the results have been inconsistent. In addition to the difficulties in collecting brain tissues from patients with MDD, the differences in studying brain areas have limited the progress of such research. Therefore, in this study, we only collected gene expression profile data from the human cortex, an important depression-related brain area, in the GEO database and conducted a series of bioinformatics analyses. Two datasets (GSE53987 and GSE54568), including 34 MDD patients and 34 healthy controls, met the defined criteria. The common differentially expressed genes in these 2 datasets were screened, and from these, 20 hub genes and related signaling pathways involved in MDD were predicted. Moreover, 10 of these hub genes showed consistent changes in the peripheral blood of MDD patients, indicating that these pathogenic genes also have diagnostic potential. Interestingly, the changes in 5 hub genes in the prefrontal cortex of depressed mice showed the same trend as those in humans, suggesting that these genes should be given more attention in future pathogenesis studies of MDD. Subsequently, based on the screened risk genes, a few traditional Chinese medicines were predicted to be effective for MDD, which may provide novel alternatives for antidepressant treatment.

## INTRODUCTION

Major depressive disorder (MDD) is a type of heterogeneous psychosomatic illness characterized by significant and persistent depression, decreased energy, and sluggish thought processes (Lei et al., 2020). This disorder is marked by high rates of morbidity, relapse, disability, and suicide and low rates of recovery (Tan et al., 2022). Since the outbreak of the new coronavirus pneumonia, the number of patients with MDD worldwide is predicted to increase by 27.6% per year, and MDD has become a global health crisis (Shader, 2020). It is anticipated that by 2030, MDD will surpass tumors and cardiovascular and cerebrovascular diseases to become the world’s leading cause of death, and its prevention and treatment will become a global priority (Lewis et al., 2019). Although it is widely acknowledged that both genetics and environmental factors play a role in the development of MDD, the molecular substrates responsible for the underlying mechanism have not been fully clarified. Consequently, relatively few effective molecular targets have been identified for the clinical diagnosis and treatment of MDD (Furukawa et al., 2019).

To facilitate such studies, some proxy tissues, such as blood, model animal tissues, and induced pluripotent stem cells, are often used to study the pathogenesis of MDD (Malhi and Mann, 2018 ; Licinio and Mazza, 2016). Inflammatory pathways, the kynurenine pathway, and growth factor–related molecular networks have been shown to be more strongly related to the etiology of MDD (Anisman et al., 2008; Mazza et al., 2020; Paul et al., 2022). However, because these tissues cannot completely imitate the complex functions of the human brain and the regulatory situation or transcriptional activities of the human brain are unique in many regards, the results obtained using these proxy samples are sometimes inconsistent with those obtained for the human brain. For example, the conflicting results found for inflammation-related signaling pathways in MDD limit clinical application (Beurel et al., 2020). Therefore, the human brain remains the most appropriate organ for studying the etiology of MDD. A few RNA sequencing studies using postmortem human brains of patients with MDD recently have been reported (Le et al., 2018; Chen et al., 2022). Nevertheless, due to extensive logistic requirements and substantial ethical and cultural hurdles, the number of brain samples used in these studies was relatively small, and this fact coupled with the different brain regions studied make the results less reproducible; moreover, few molecules have been found to have potential for MDD diagnosis and treatment (Liu et al., 2020). Collecting more brain tissue samples and conducting transcriptome analysis of specific depression-related brain regions are effective strategies for resolving the abovementioned problems, but these studies will take more time, effort, and money. Alternatively, collecting and combining some publicly available human brain data for reanalysis may be convenient and effective.

Therefore, in this study, some mRNA microarray datasets were collected from the Gene Expression Omnibus (GEO), and the differentially expressed genes (DEGs) in specific brain regions between the MDD and control groups were screened. The DEGs were subjected to Gene Ontology (GO) functional annotation analysis and Kyoto Encyclopedia of Genes and Genomes (KEGG) pathway enrichment analysis. MDD-related hub genes and their regulatory network were then identified through a series of bioinformatics analyses. The value of these hub genes in MDD diagnosis, their involvement in MDD pathogenesis, and their potential in anti-MDD herb prediction were then assessed. This study may not only deepen our understanding of the etiology of depression but may also provide some new ideas for its diagnosis and treatment.

## MATERIALS AND METHODS

### Animals

SPF-grade C57BL/6J male mice (Vital River Laboratories, Beijing, China, aged 8–10 weeks, body weight of 22 ± 2 g) were raised by special personnel in the animal room. All operations were in accordance with the management and ethics regulations of Shandong First Medical University on the use of experimental animals.

### Establishment of Depression Models

Ten mice were assigned to each of the 2 groups in this study: normal control (NC) group and chronic unpredictable mild stress (CUMS)-treated group. The mice in the CUMS group were randomly subjected to the following stressors: cage tilting for 24 hours, swimming in cold water at 6°C for 5 minutes, water or food deprivation for 24 hours, level shaking for 15 minutes, tail nip for 1 minute, 45°C heat stress for 5 minutes, and inversion of the light/dark cycle for 24 hours. The same stressor was not given in succession.

### Animal Behavior Analysis

#### Forced swimming test (FST)

The mice were forced to swim for 6 minutes in a transparent plastic 15 cm of water (water temperature maintained at 22°C –23°C), and the immobility time over the last 4 minutes was recorded. At the end of the experiment, the mice were removed and heated under a heat lamp until their bodies were dry.

**Sucro** **s** **e pre** **ference** **test** **(SPT)**

After mice were acclimated to 2 drinking bottles for 3 days, each mouse was isolated in individual cages, and 2 drinking bottles were placed in each cage (one with 1% sucrose and the other containing water) to measure the volume of intake of water and sucrose solution. The formula was calculated as follows. Sucrose preference (expressed as %) = V (sucrose solution)/[V (sucrose solution) + V (water)] × 100.

### RNA Extraction and Quantitative Real Time Polymerase Chain Reaction (qRT-PCR)

After the behavioral test, the prefrontal cortices of the mice in the CUMS and NC groups were precisely dissected and then stored in a −80°C freezer for detection.

TRIzol was used for the extraction total RNA from the mouse prefrontal cortex. The reaction system was prepared using the SYBR quantitative PCR kit (TB Green Premix Ex Taq, Takara), and the reaction was conducted in a qPCR instrument (LightCycler96, Roche). The 2^−ΔΔCt^ relative quantification method was used to examine the transcript levels of the genes to be tested, and actin was used as an internal reference. Table 1 shows the primer sequences for each gene.

**Table 1.** Primer Sequences

Gene	Forward (5’ → 3’)	Reversed (5’ → 3’)
Smarca4	TGGCACCAAGACACTGATGAACAC	CTGAGGCACGGTAAAGGTCCAATC
Kdm6b	TGAAGAACGTCAAGTCCATTGTG	TCCCGCTGTACCTGACAGT
Smarcc2	AACCGCCAACCAACAAGTCT	AGGAAACATTTGATCGGCAGT
Arid1b	CGTGCGGAGCTTGTCTTTC	CCTCCTTCTCATAGGTCTGTGG
Scaf11	GTGGATGAGACAGGAGGAGGAGAC	CGTTCACTGGAGGCTGCATCAC
Srsf11	GTCCCTGATTTCTGCCGCTATTGAG	AGTGTGACCGCCTCCTTGACC
Thoc2	CAAGAGAGCACCGAAGCAAGTAGAG	TGAGAGGTGAGGACCAAGGTAACAG
Cux2	AGAAGGAGGCTCTGCGGAAGG	CGTCTTGAGGTTGAGTTGGAAGGAG
Naaa	CTGAATGGAGAGTGGTTCCGAGTTG	TTAGGGCTTTGATGGCTGGTGTTC
Phkb	TCACAACCGCAACAGGCAGAC	ATCCAAGGCAAACGCAGGGTAAC
Actb	GGCTGTATTCCCCTCCATCG	CCAGTTGGTAACAATGCCATGT
Pten	GGAAAGGGACGGACTGGTGTAATG	CGCCTCTGACTGGGAATTGTGAC
Mapk1	GCCTTCCAACCTCCTGCTGAAC	CGTACTCTGTCAAGAACCCTGTGTG
Nfya	AGACAGGAGCCAATACCAACACAAC	GGGATTCTTTGGATAGCAGGCACAG
Gtf2h1	ACACAGCAAGCCGTAAACCAGATG	AAGTTCCCCAACAGCCACATACAAG
Crk	CAGTGGTGGAATGCAGAGGACAG	AGGCGGAGGCAGGTCTATACTTC
Ccng2	CACTTGGCAGGTGGCGAAGG	CCCGAGGTTGGTATCTCTGTTCTTG
Acer3	GGTGACCTTGTTCGTCGCTGAG	CGCTTCTCCAGTCTGTCTCTAATGC
Slc4a2	AGCAGCAGCAGCAGCAATATGAC	CCAGCCATTAGCACCAGCGATAG
Rhoa	CGGAATGACGAGCACACGAGAC	TCCTGTTTGCCATATCTCTGCCTTC

### Data Collection

We conducted a systematic search of the public GEO database (https://www.ncbi.nlm.nih.gov/geo/) (Clough and Barrett, 2016) for gene expression profile datasets from the prefrontal cortex of MDD patients and controls. The inclusion criteria were as follows: (1) only from the GPL570 platform; (2) diagnosed with MDD; (3) the data were obtained after 2010 and up to October 2022; and (4) ≥15 patients. GSE53987 contains information from 19 MDD patients and 19 healthy controls. Brain tissues were collected separately from the dorsolateral prefrontal cortex, dorsal striatum, and hippocampus (Lanz et al., 2019). The GSE54568 dataset contains gene expression profiles from the bilateral dorsolateral prefrontal cortex of 15 patients with MDD and 15 controls (Chang et al., 2014). The data analyzed in this experiment were obtained from public databases and did not require patient consent or ethics committee approval.

### DEG Screening

The raw matrix file data of GSE53987 and GSE54568 were downloaded, and the platform’s gene probes were converted into gene names with reference to the GPL570 platform. Data normalization and DEG screening were performed using the online analysis tool GEO2R. The Benjamini and Hochberg method for controlling the false discovery rate was employed to eliminate false-positive findings, and the resulting *P* values were computed. To identify genes with significant differences in the expression fold change (FC), we set the following screening criteria for filtering genes: FC value (|log2FC|) >1 and *P* < .05. The online tool Venn 2.1 was employed to generate a Venn diagram of the identified DEGs, and a heatmap of the DEGs was drawn using Heml Software.

### Functional Enrichment Analysis

To enable better recognition of the biological functions of DEGs coexisting in the GSE53987 and GSE54568 datasets, we performed GO enrichment and KEGG analyses using online tools. Based on the description of the GO analysis, the gene function annotations were classified as biological processes (BPs), cellular components (CCs), or molecular functions (MFs). DAVID (https://david.ncifcrf.gov/) (Huang et al., 2009) and KOBAS (version 3.0; https://bio.tools/kobas) (Mao et al., 2005) are versatile bioinformatics tools. Functional annotation analysis of the common DEGs was performed using DAVID, and KEGG signaling pathway analysis of the common DEGs was performed using KOBAS. *P* < .05 was considered to indicate statistical significance.

### Protein–Protein Interaction (PPI) Network Analysis

To predict PPIs, STRING, an online database that can retrieve the interactions between a group of proteins, was utilized for PPI network analysis (Szklarczyk et al., 2021). To determine protein interrelationships, we uploaded the common DEGs from the GSE53987 and GSE54568 datasets to STRING’s official website. The minimum required interaction score was set to 0.7, and the interaction networks were visualized with Cytoscape (version 3.7.1) (Shannon et al., 2003). Molecular Complex Detection (MCODE), an APP of Cytoscape, was then used to select the most significant PPI network modules. CytoHubba was used to identify important nodes, which were then used to select key genes using a degree algorithm, and the top 20 genes were designated as key genes. We overlapped key network modules identified by MCODE with key genes obtained from cytoHubba to identify hub genes.

### Potential of Hub Genes for MDD Diagnosis

To assess the potential of the identified hub genes for the diagnosis of MDD, an additional public database, which contains peripheral blood transcriptome data from 161 depressed patients and 169 control patients (Zhao et al., 2021), was used for further analysis. The expression levels of the identified hub genes in different groups were downloaded and analyzed.

### Circle RNA (circRNA) - MicroRNA (miRNA) - Messenger RNA (mRNA) Network

To identify the posttranscriptional regulatory mechanisms of the identified hub genes, LncRNADisease database 2.0 (http://www.rnanut.net/lncrnadisease/) (Bao, et al., 2019), starBase database (http://starbase.sysu.edu.cn/) (Li et al., 2014), and miRNet (http://www.mirnet.ca/) (Chang et al., 2020) were used. First, we used the term “major depressive disorder” to search the lncRNADisease database to find circRNAs associated with MDD, entered the identified circRNAs in the starBase database to filter out the corresponding miRNAs, used miRNet to find related mRNAs that bind with the obtained miRNAs, and finally intersected these mRNAs with the screened hub genes. The regulatory network was then visualized using Cytoscape software (version 3.7.1).

### Prediction of Traditional Chinese Medicines (TCMs) for Treatment of MDD

Coremine Medical is an advanced medical information retrieval platform (Kong et al., 2017 ). The screened hub genes were entered into the Coremine Medical search box, and *P* <.05 was used as the criterion for the screening of TCMs. The herbs that corresponded to each hub gene were predicted for further analysis and discussion.

### Statistical Analysis

Data analysis was performed using GraphPad Prism (9.0.0, USA). Continuous variables are presented as the means ± SDs. The means of multiple groups were compared by 1-way ANOVA. The results from qPCR-derived gene expression experiments, FST and SPT were analyzed by paired Student’s *t* test for 2-group comparisons. Spearman coefficients were used to assess the correlations between genes. All statistical *P* values calculated were 2-sided, and *P *< .05 was considered to indicate statistical significance.

## RESULTS

### DEGs in Prefrontal Cortex of MDD Patients

**Figure 1.**
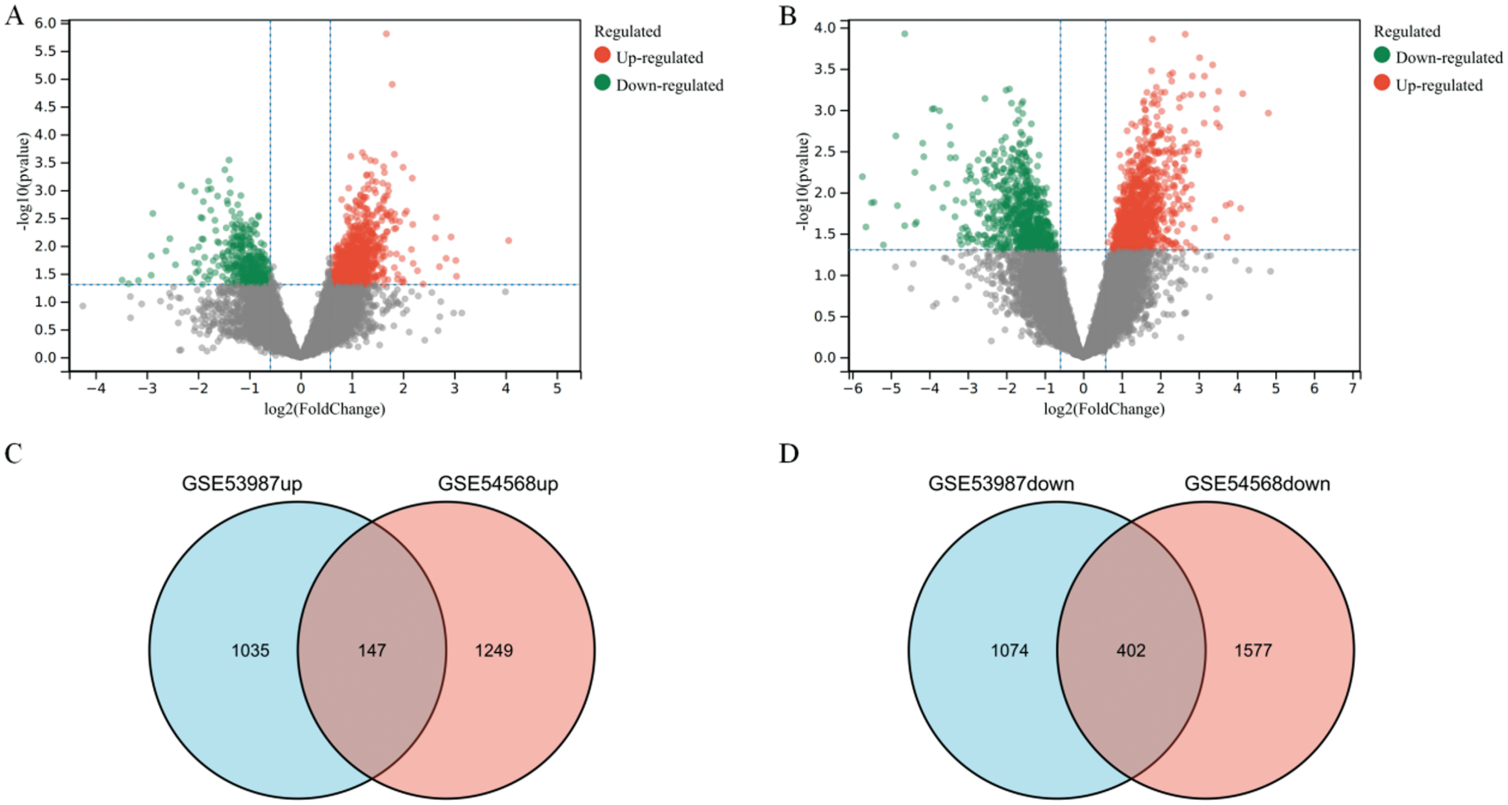
Differentially expressed RNAs in major depressive disorder (MDD). (A) Volcano plot of differentially expressed mRNAs in GSE53987. (B) Volcano plot of differentially expressed mRNAs in GSE54568. (C) Venn diagram shows the common upregulated genes in the 2 datasets. (D) Venn diagram shows the common downregulated genes in the 2 datasets.

GSE53987, GSE54568, and GSE44593 are the 3 datasets that meet the criteria established in this study. However, because GSE44593 data were derived from a gene expression analysis of whole brain tissue and were not highly specific, this dataset was excluded from the following study. GEO2R was used to analyze the GSE53987 and GSE54568 datasets, and the upregulated and downregulated genes in the MDD group compared with the control group in each dataset were filtered based on the criteria |Log2FC| >1 and *P *< .05 (Figure 1). The results obtained with the GSE53987 dataset identified 2658 DEGs (1182 upregulated genes and 1476 downregulated genes in the prefrontal cortex of MDD patients compared with controls) (Figure 1A). GSE54568 contained 3375 DEGs (1396 upregulated genes and 1979 downregulated genes) (Figure 1B). The intersection of upregulated and downregulated genes in the 2 datasets contained 147 upregulated and 402 downregulated genes, respectively (Figure 1C–D). These DEGs common to the GSE53987 and GSE54568 datasets were defined as the candidate genes to be analyzed in the following studies.

### GO Function and KEGG Pathway Enrichment Analyses of DEGs

**Figure 2.**
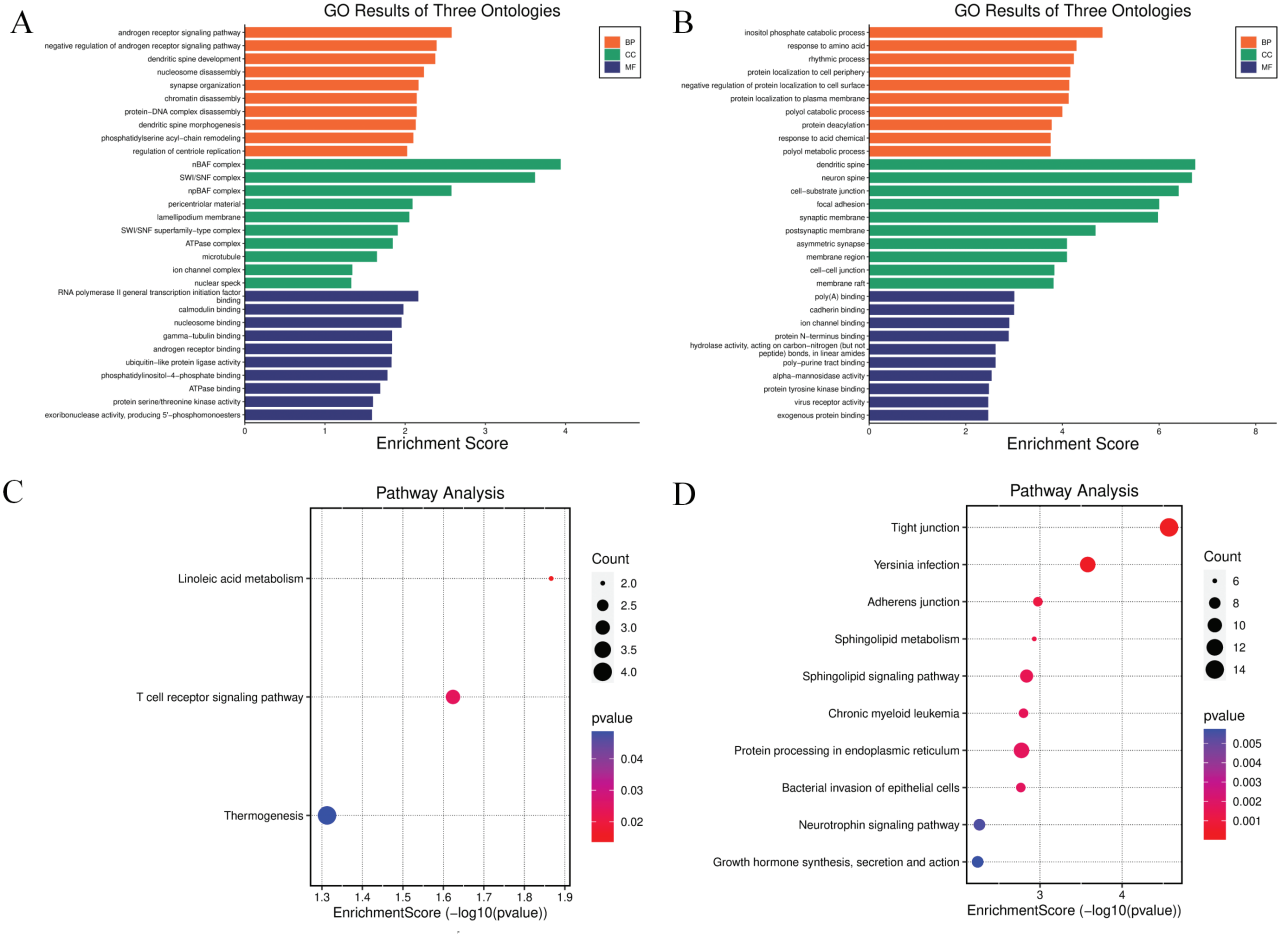
The gene ontology (GO) functions and Kyoto encyclopedia of genes and genomes (KEGG) pathways of the upregulated and downregulated differentially expressed genes (DEGs). (A) GO function enrichment map of the upregulated DEGs. (B) GO function enrichment map of the downregulated DEGs. (C) KEGG pathway enrichment map of upregulated DEGs. (D) KEGG pathway enrichment map of downregulated DEGs.

To gain a more comprehensive understanding of the selected DEGs, GO function annotation and KEGG pathway enrichment analyses were performed. Within the BP category, the upregulated DEGs were primarily associated with the androgen receptor signaling pathway, negative regulation of androgen receptor signaling pathway, dendritic spine development, nucleosome disassembly, and synapse organization. The barrier-to-autointegration factor (nBAF) complex, SWI/SNF complex, npBAF complex, pericentriolar material, and lamellipodium membrane were the CC terms enriched in DEGs. In terms of MFs, the DEGs were mainly enriched in RNA polymerase II general transcription initiation factor binding, calmodulin binding, nucleosome binding, gamma-tubulin binding, and androgen receptor binding (Table 2; Figure 2A). The analysis of BPs revealed that the downregulated DEGs were mainly involved in the inositol phosphate catabolic process, response to amino acid, rhythmic process, protein localization to cell periphery, and negative regulation of protein localization to cell surface. Dendritic spine, neuron spine, cell-substrate junction, focal adhesion, and synaptic membrane were identified as the enriched CC terms, and poly(A) binding, cadherin binding, ion channel binding, protein N-terminus binding, and hydrolase activity, acting on carbon-nitrogen (but not peptide) bonds, in linear amides were the enriched MFs (Table 2; Figure 2B).

**Table 2.** Gene Ontology (GO) Enrichment Analysis of Screened Common Differentially Expressed Genes (DEGs)

GO term	Description	*P* value	Q value	Count
BP	Regulation of dendritic spine development	2.23091E-05	0.05841006	10
	Dendritic spine development	4.54991E-05	0.05841006	11
	Inositol phosphate catabolic process	6.03024E-05	0.05841006	5
	Regulation of transmembrane transporter activity	6.43521E-05	0.05841006	19
	Synapse organization	8.81996E-05	0.05841006	25
	Regulation of ion transmembrane transporter activity	.000118068	0.05841006	18
	Regulation of transporter activity	.000129366	0.05841006	19
	Regulation of dendrite development	.00013314	0.05841006	13
	Dendritic spine morphogenesis	.000141072	0.05841006	8
	Dendrite development	.000154838	0.05841006	17
CC	Dendritic spine	4.16561E-07	0.00011218	18
	Neuron spine	4.93384E-07	0.00011218	18
	Synaptic membrane	1.70251E-06	0.000258065	27
	Cell-substrate junction	2.49249E-05	0.002444504	26
	Postsynaptic membrane	2.68782E-05	0.002444504	20
	Focal adhesion	4.94253E-05	0.003612314	25
	Asymmetric synapse	5.56062E-05	0.003612314	22
	Neuron to neuron synapse	.000154431	0.008778174	22
	Postsynaptic specialization	.000312965	0.01519871	21
	Postsynaptic density	.000334231	0.01519871	20
MF	Ion channel binding	.000189549	0.125900576	12
	Phosphatidylinositol-4-phosphate binding	.00101342	0.242145518	5
	Protein serine/threonine kinase activity	.001244292	0.242145518	23
	Protein N-terminus binding	.002035032	0.242145518	9
	Hydrolase activity, acting on carbon-nitrogen (but not peptide) bonds, in linear amides	.002186099	0.242145518	7
	ATPase binding	.002187368	0.242145518	8
	Poly(A) binding	.002798603	0.259040581	4
	Cadherin binding	.003119982	0.259040581	18
	ATPase regulator activity	.004207567	0.273438156	5
	Nuclear receptor binding	.005124756	0.273438156	8

BP, biological process; CC, cellular component; MF, molecular function

The KEGG pathway enrichment analysis demonstrated that the upregulated DEGs were mainly involved in linoleic acid metabolism, the T-cell receptor signaling pathway, and thermogenesis (Table 3; Figure 2C). The downregulated DEGs were mainly enriched in tight junctions, protein processing in the endoplasmic reticulum, adherent junctions, sphingolipid metabolism, and the sphingolipid signaling pathway (Table 3; Figure 2D).

**Table 3.** Kyoto Encyclopedia of Genes and Genomes (KEEG) Enrichment Analysis of Screened Common differentially expressed genes (DEGs)

ID	Description	*P* value	Q value	Count
hsa04530	Tight junction	.000315522	0.067197673	14
hsa05135	Yersinia infection	.000510702	0.067197673	12
hsa00600	Sphingolipid metabolism	.002503831	0.136215092	6
hsa04014	Ras signalling pathway	.002518465	0.136215092	15
hsa04141	Protein processing in endoplasmic reticulum	.003458265	0.136215092	12
hsa04261	Adrenergic signalling in cardiomyocytes	.003612436	0.136215092	11
hsa04520	Adherens junction	.003910695	0.136215092	7
hsa04510	Focal adhesion	.004788908	0.136215092	13
hsa05220	Chronic myeloid leukemia	.005704752	0.136215092	7
hsa05100	Bacterial invasion of epithelial cells	.006127249	0.136215092	7

### PPI Network Construction and Hub Gene Identification Based on DEGs

**Figure 3.**
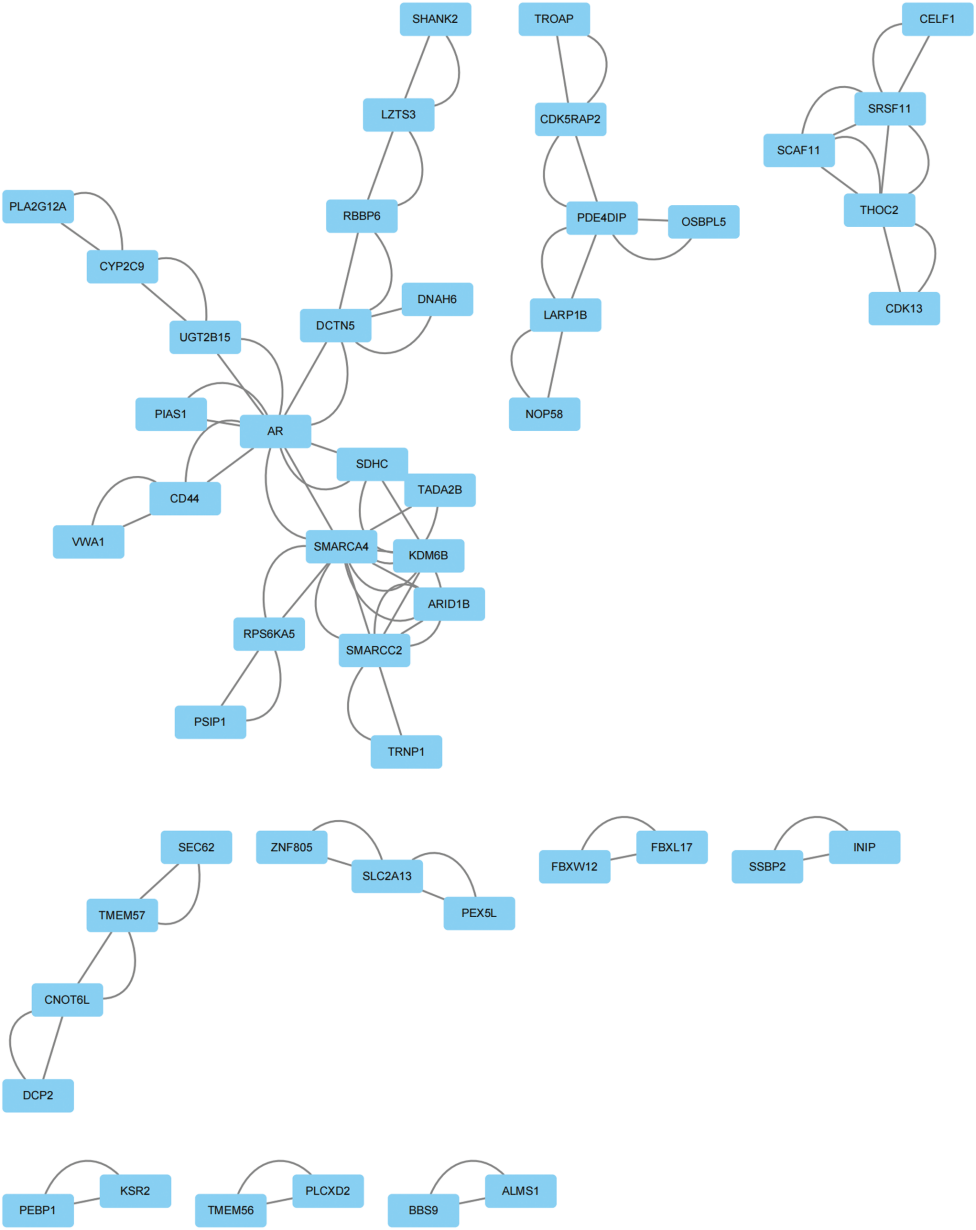
Protein-protein interaction (PPI) network constructed from the upregulated differentially expressed genes (DEGs).

**Figure 4.**
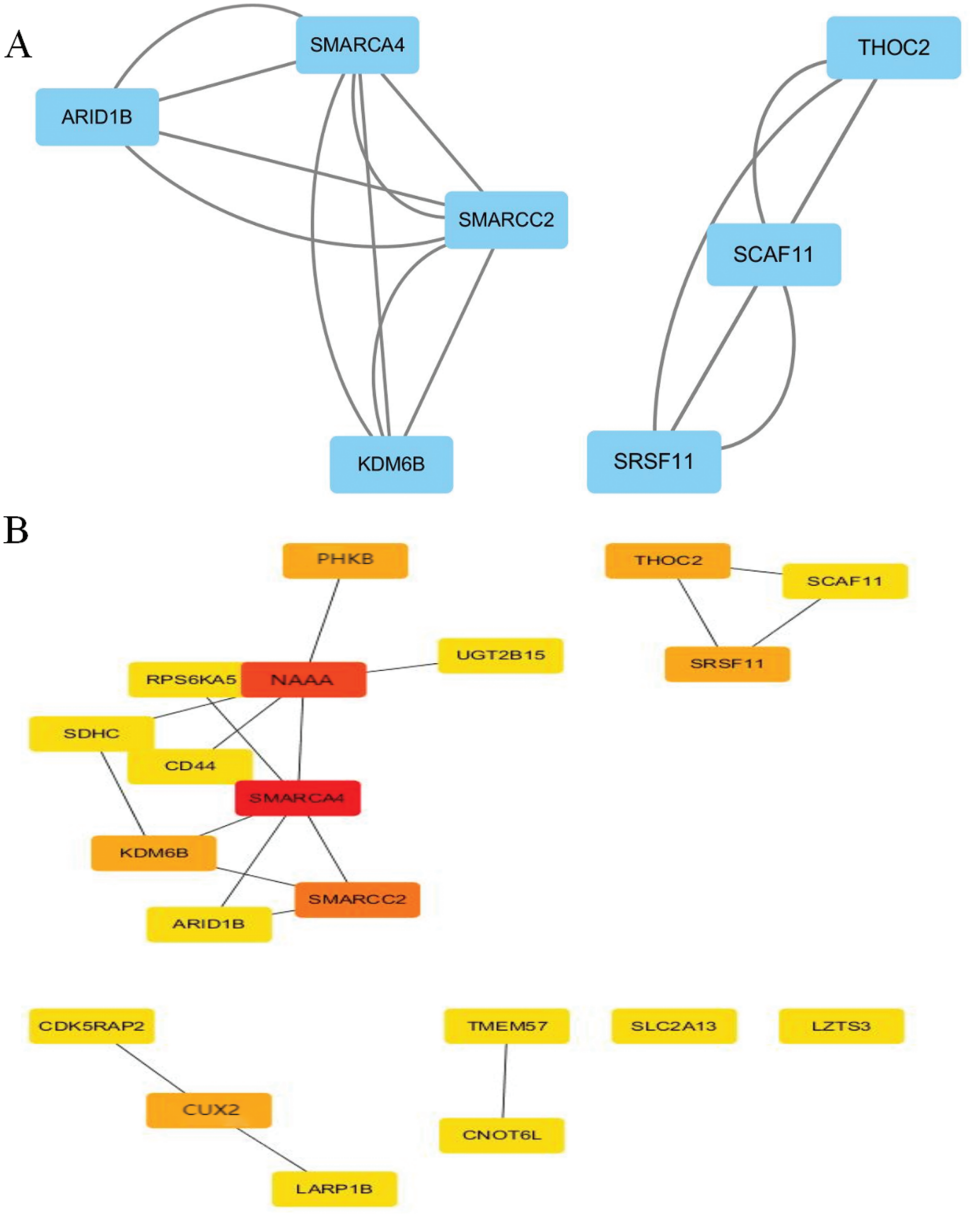
Protein-protein interaction (PPI) network construction and hub gene identification from the upregulated differentially expressed genes (DEGs). (A) Using MCODE plug-in to screen out the most significant modules from the PPI network. (B) Using Cytohubba plug-in to screen the top 20 key genes in the most significant modules.

**Figure 5.**
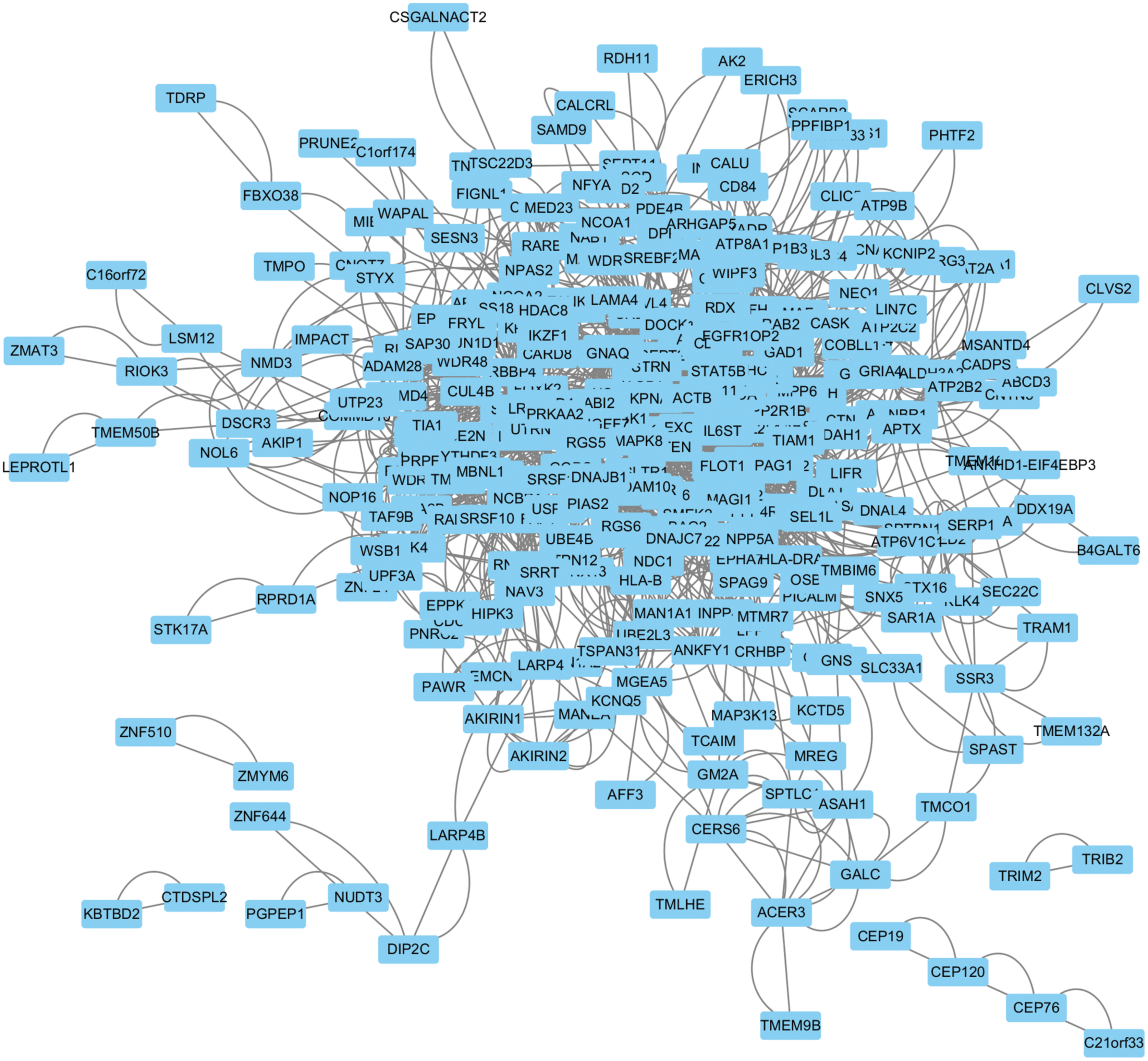
Protein-protein interaction (PPI) network constructed from the downregulated differentially expressed genes (DEGs).

**Figure 6.**
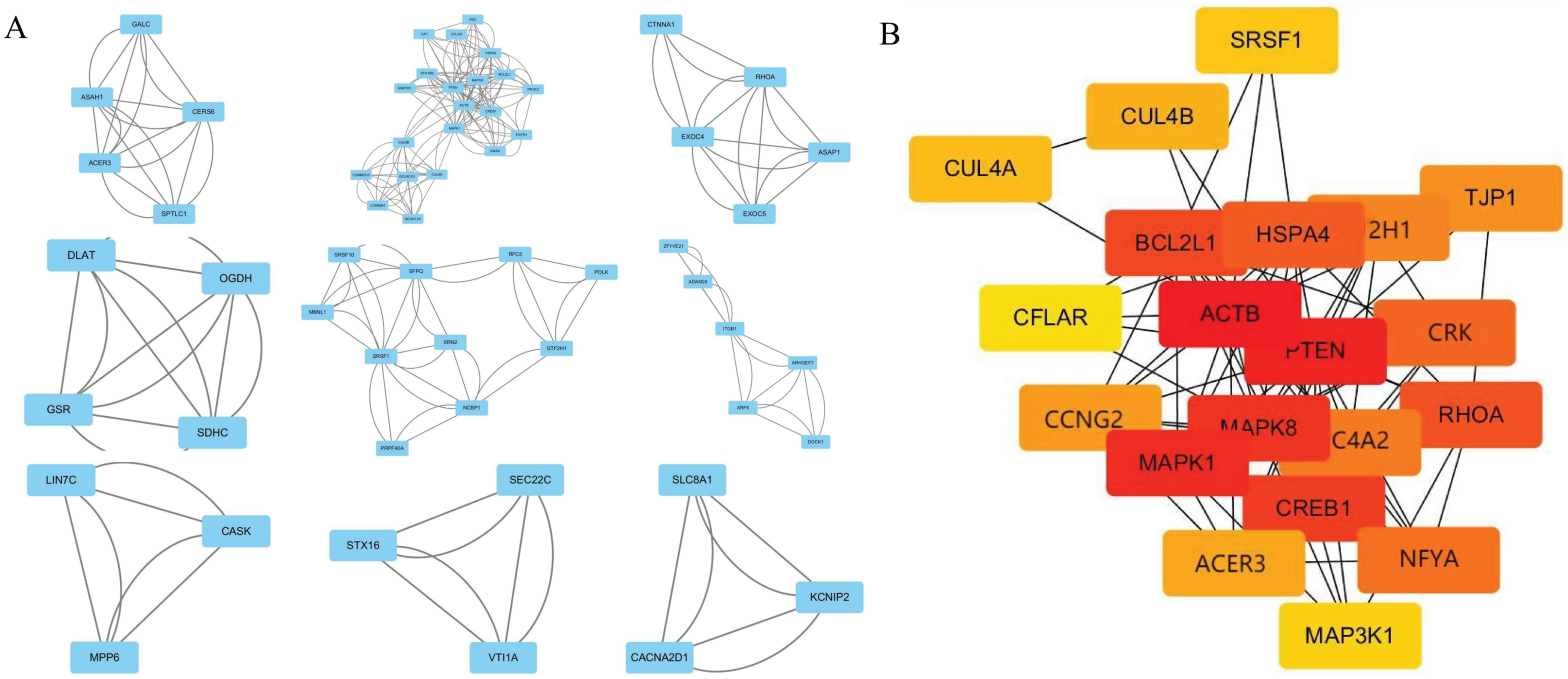
Protein-protein interaction (PPI) network construction and hub gene identification from the downregulated differentially expressed genes (DEGs). (A) Using MCODE plug-in to screen out the most significant modules from the PPI network. (B) Using Cytohubba plug-in to screen the top 20 key genes in the most significant modules.

To systematically analyze the interaction relationship of the identified DEGs, a PPI analysis of the 147 upregulated genes was first performed with the STRING database. The TSV file of the network was downloaded and imported into Cytoscape software for visualization. A total of 87 nodes and 460 edges were obtained (Figure 3). Two modules with the most significant interactions in the PPI network graph were obtained using Cytoscape’s MCODE plug-in, and these modules contained 7 nodes and 16 edges (Figure 4A). The MNC algorithm, a CytoHubba plug-in of Cytoscape, was used to identify the top 20 key genes according to the total score (Figure 4B), and a darker node color indicates a higher gene score. Combining the above 2 calculation methods, we identified *SMARCA4*, *KDM6B*, *SMARCC2*, *ARID1B*, *SCAF11*, *SRSF11*, *THOC2*, *CUX2*, *NAAA*, and *PHKB* as the hub genes among the upregulated genes. Similarly, for the 402 downregulated genes, we also used the STRING database for PPI analysis and Cytoscape software for visualization (Figure 5). Using the MCODE plug-in of Cytoscape, the analysis revealed that 9 modules, which had a total of 60 nodes and 268 edges, interacted significantly (Figure 6A). We also chose the top 20 key genes after analysis using cytoHubba (Figure 6B). Combining the above 2 calculation methods, we identified *ACTB*, *PTEN*, *MAPK1*, *NFYA*, *GTF2H1*, *CRK*, *CCNG2*, *ACER3*, *SLC4A2*, and *RHOA* as the hub genes among the downregulated genes.

### Potential of Screened Hub Genes for Diagnosis of MDD

**Figure 7.**
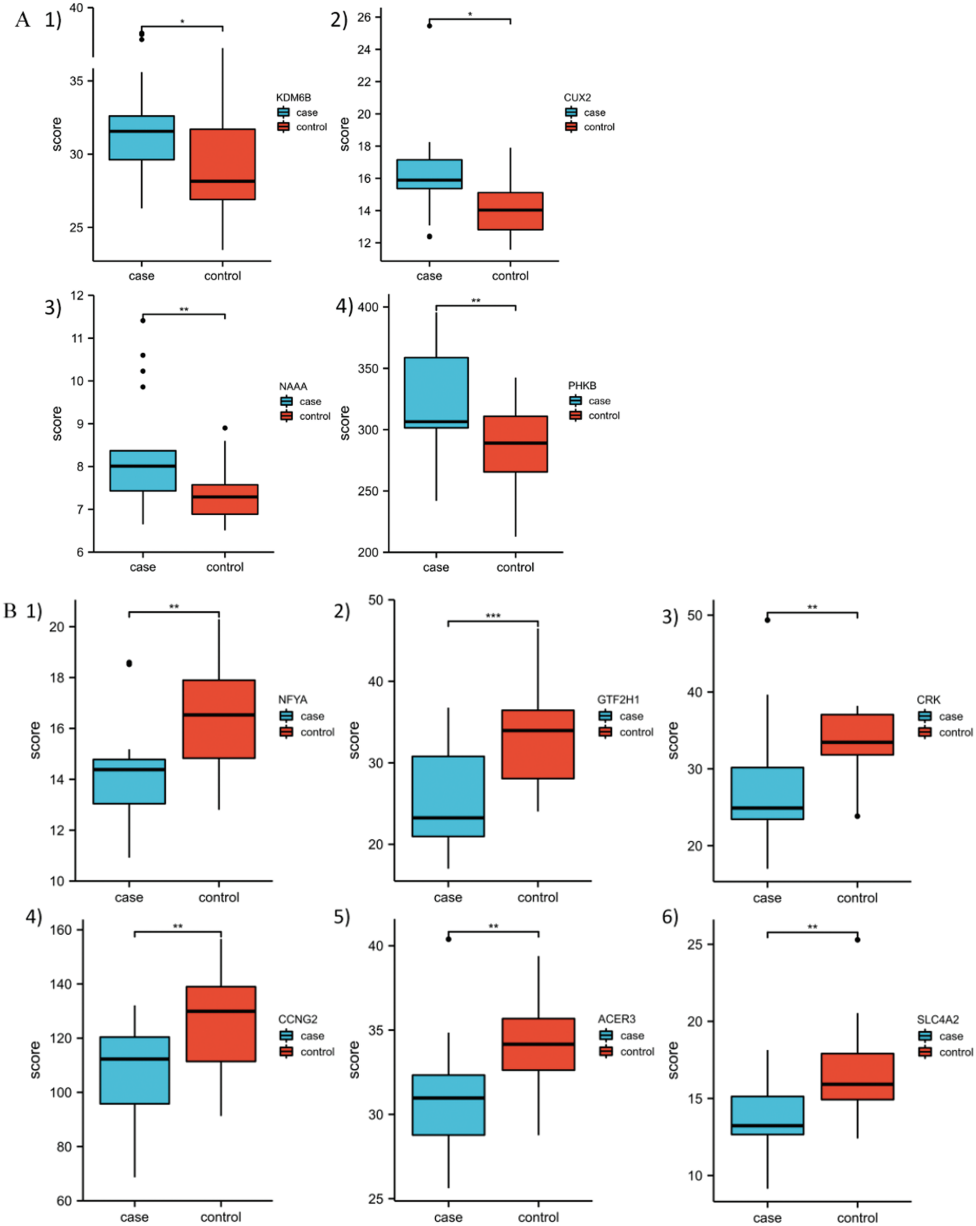
Expression of screened hub differentially expressed genes (DEGs) in the peripheral blood of major depressive disorder (MDD) patients. (A) Among the upregulated hub genes, *KDM6B*, *CUX2*, *NAAA*, and *PHKB* also showed markedly higher expression in the blood of MDD patients. (B) Among the downregulated hub genes, *NFYA*, *GTF2H1*, *CRK*, *CCNG2*, *ACER3*, and *SLC4A2* also exhibited significantly lower expression in the blood of MDD patients. *P < .05, **P < .01, ***P < .001.

Subsequently, we sought to explore whether the identified hub genes are useful for the diagnosis of MDD by analyzing a cohort containing transcriptomic data from whole-blood samples of 161 MDD patients and 169 controls. The results showed that among these hub genes, *KDM6B*, *CUX2*, *NAAA*, and *PHKB* showed markedly higher expression in the blood of MDD patients (Figure 7A), whereas *NFYA*, *GTF2H1*, *CRK*, *CCNG2*, *ACER3*, and *SLC4A2* exhibited significantly lower expression (Figure 7B), which was consistent with the changes in the expression of these hub genes in the prefrontal cortex of MDD patients. This finding suggests that some hub DEGs, including *KDM6B*, *CUX2*, *NAAA*, *PHKB*, *NFYA*, *GTF2H1*, *CRK*, *CCNG2*, *ACER3*, and *SLC4A2*, may be potential biomarkers for the diagnosis of MDD, and a larger sample size and more rigorous experimental design are needed for verification in the future.

### Involvement of Screened Hub Genes in Occurrence of Depressive-Like Behaviors in Mice

**Figure 8.**
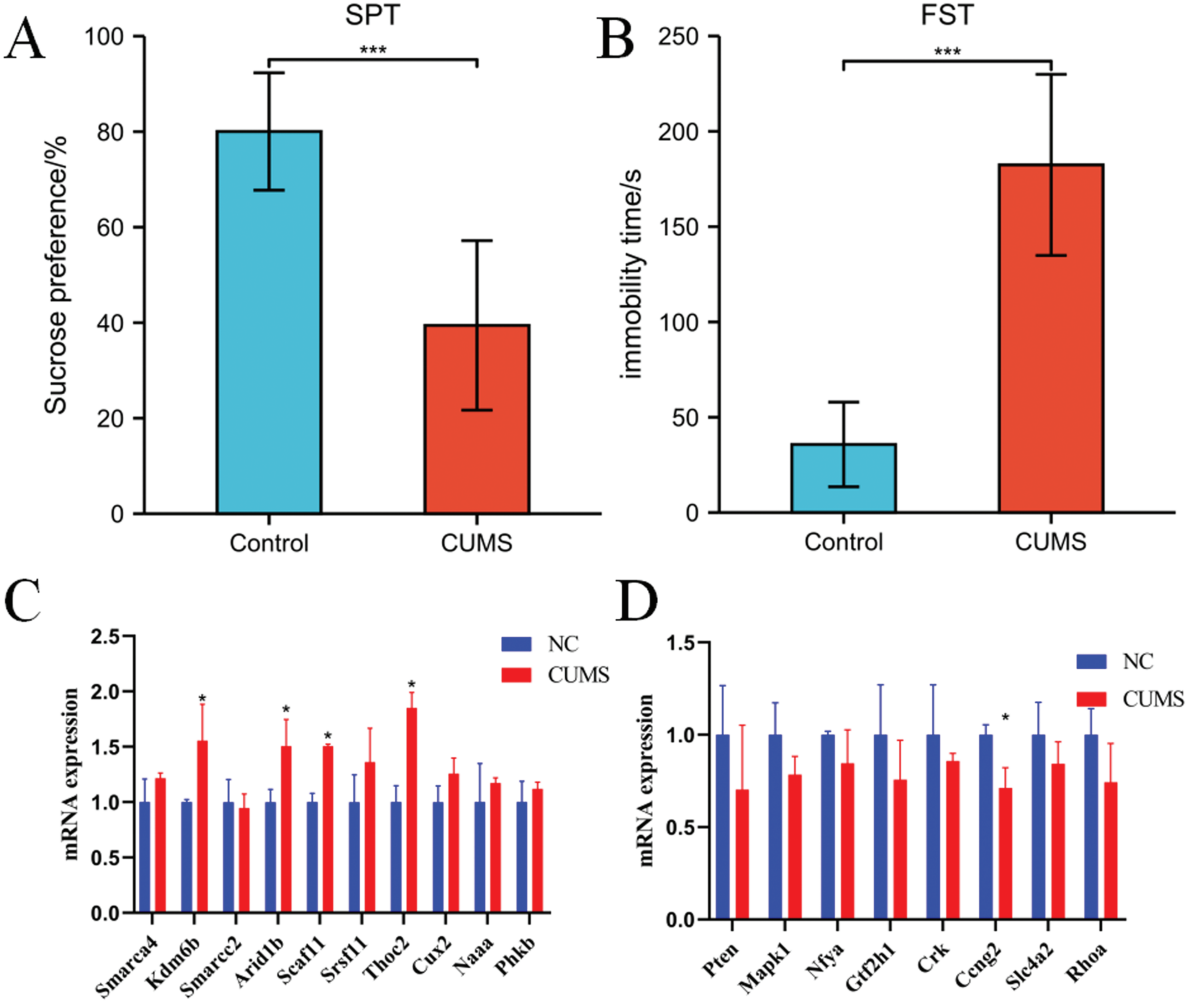
Expression of screened hub differentially expressed genes (DEGs) in the brains of mice with depressive behaviors. (A) After 4 weeks of cmhronic unpredictable ild stress (CUMS) treatment, the sugar water preference value of the mice was significantly decreased in the sucrose preference test (SPT) compared with that of the normal control (NC) group. (B) After 4 weeks of CUMS treatment, the immobility duration of the mice was significantly extended in the forced swimming test (FST) compared with that of the NC group. (C) The expression of upregulated hub genes in the prefrontal cortex of CUMS mice and their controls using quantitative real time polymerase chain reaction (qRT-PCR) assay. (D) The expression of downregulated hub genes in the prefrontal cortex of CUMS mice and their controls using RT-PCR assay. *P < .05, ***P < .001.

CUMS may induce some depressive-like behaviors in rodents, and the resulting model is commonly used to mimic the pathophysiology of depression. Based on this model, the detailed involvement of some genes in the pathogenesis of MDD can be fully studied. To evaluate whether the screened hub genes play roles in the occurrence of MDD, their expression changes in the prefrontal cortex of CUMS-treated mice were detected. After 4 weeks of CUMS treatment, the sugar water preference value of the mice was significantly decreased in the SPT (*P* < .001; Figure 8A), whereas the immobility duration in the FST was significantly extended (*P* < .001; Figure 8B) compared with that of the NC group. These findings revealed that mouse models with depression-like behaviors were generated effectively. The prefrontal cortex tissues were collected, and RT-PCR was performed to detect the expression of 20 hub genes in the CUMS and NC groups. The results indicated that the mRNA expression of *Kdm6b*, *Arid1b*, *Scaf11*, and *Thoc2* in the prefrontal cortex tissue of the mice in the CUMS group was higher than that of the mice in the NC group (Figure 8C), whereas the mRNA expression of *Ccng2* in the prefrontal cortex tissue of the CUMS group was significantly lower than that of the NC group (Figure 8D), which was consistent with the expression changes in the human cerebral cortex. This finding shows that some of the screened hub genes exhibit brain region–specific expression changes in a mouse model of depression like those observed in the human brain, which indicates that these genes should be given more attention in subsequent studies of the pathogenesis of MDD.

### Construction of circRNA-miRNA-mRNA Regulatory Network Targeting the Screened Hub Genes

**Figure 9.**
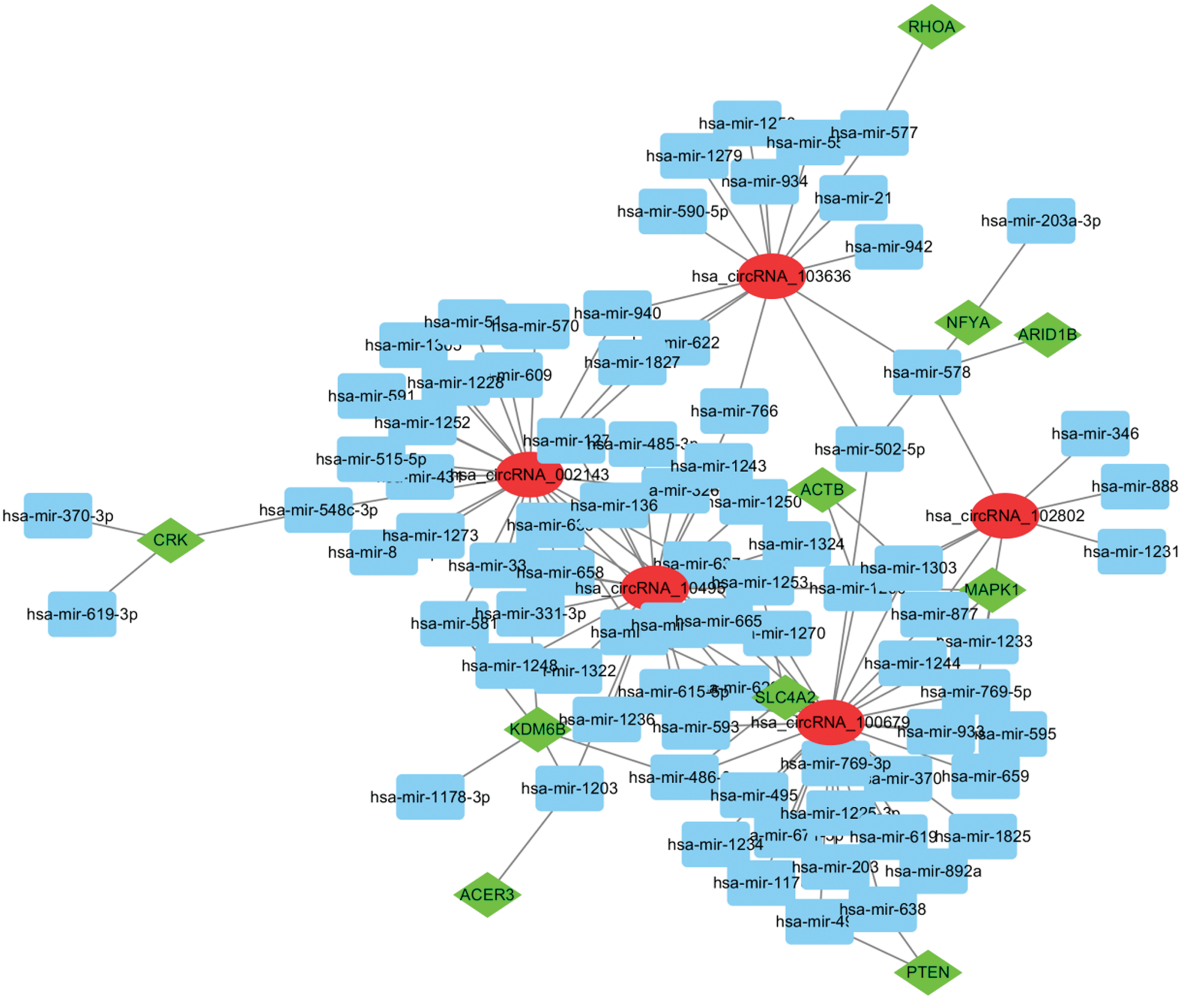
Circle RNA (circRNA) - MicroRNA (miRNA) - Messenger RNA (mRNA) regulatory network of major depressive disorder (MDD)-related hub genes. Circles, rectangles, and diamonds respectively represent differentially expressed circRNAs, miRNAs, and mRNAs.

Posttranscriptional RNA regulation is an important process for controlling gene expression. A growing body of evidence shows that circRNA-related competitive endogenous RNA (ceRNA) regulatory networks play a crucial role in the expression of mRNAs (Zhou et al., 2020). To further elucidate the circRNA-dependent regulatory mechanism of MDD-related genes, the 5 most substantially changed circRNAs were first identified using the LncRNADisease database, and these circRNAs were hsa_circRNA_002143, hsa_circRNA_100679, hsa_circRNA_102802, hsa_circRNA_103636, and hsa_circRNA_104953. A search of the StarBase database then identified 77 miRNAs corresponding to the 5 circRNAs. The target genes of these 77 miRNAs were predicted using miRNet, which yielded a total of 6808 genes. The intersection of these target genes with the discovered hub genes revealed 10 common genes. Using Cytoscape, a circRNA-miRNA-mRNA network, which included 10 differentially expressed mRNAs, 77 miRNAs, and 5 circRNAs, was constructed (Figure 9). These regulatory networks might be helpful for clarifying the regulatory mechanisms upstream of the hub genes in the future.

### Anti-MDD Herb Prediction Based on Screened Hub Genes

TCM has a long history in treating MDD. Coremine Medical is an advanced medical information retrieval platform that can be used to predict TCMs for MDD treatment according to specific genes (Kong et al., 2017). Therefore, we next prioritized the aforementioned hub genes for further TCM prediction. A total 140 TCMs related to the 20 hub genes were obtained (Table 4). Among these TCMs, citron, fructus citri, leaves of *Panax notoginseng*, sanchi flower, pseudoginseng, and dan-shen root were predicted to concurrently affect at least 3 hub genes, which suggests that these TCMs might be potential therapeutic candidates for MDD and that further research is needed to identify and confirm the anti-MDD active ingredients of these TCMs.

**Table 4.** TCM Prediction Associated With Hub DEGs in MDD

Gene	Predictive Traditional Chinese Medicine
kdm6b	Lizard (0.00113)
smarcc2	*Levant Cotton Root* (0.00288), *Cottonseed* (0.00624), *Corn Stigma* (0.0379)
srsf11	*Asparagus* (0.00502), *Purple Perilla* (0.0154)
cux2	*Citron* (0.00148), *Fructus Citri* (0.00151)
naaa	Rhizoma Atractylodis (0.00117), Grain Bud (0.00321)
actb	Musk (0.0153), *Huperzia Serrata* (0.026), *Caulis Sinomenii* (0.027), *Glossy Ganoderma* (0.0344), Chinese-date (0.0407), Arnebia guttata Bunge (0.0409), Utricularia Bifida (0.00655), Fennel flower Seed (0.0105), *Dendrobium loddigesii Rolfe* (0.0121), Curcuma Longa (0.0149), Rhizoma Curcumae Longae (0.015), Common Sage (0.0168), *Alpinia Oxyphylla* (0.0213), Garcinia (0.0239), *Rhizoma Sparganii* (0.0273), Smilax Glabra Rhizome (0.0291), Creeping Oxalis (0.0291), Leaves of Panax Notoginseng (0.0326), Sanchi Flower (0.0326), Pseudoginseng (0.0349), *Polygonatum Cyrtonema Hua* (0.0364), Dan-Shen Root (0.0387), Cactus (0.0484)
mapk1	*Ophioglossum Vulgatum* (0.00167), *Boswellia Carterii* (0.00191), Purple Bergenia Herb (0.00294), Hemerocallis Fulva Root (0.00315), *Sculellaria Barbata* (0.00551), *Oldenlandia* (0.006), *Chinese Lobelia* (0.0072), *Semen Cuscutae* (0.00796), *Longspur Epimedium* (0.00908), *Lytta* (0.00914), *Sargentodoxa Cuneata* (0.00914), *Smilax Glabra Rhizome* (0.0109), *Siebold Wildginger herb* (0.0109), Small Structure Bark (0.0113), *Bolbostemma Paniculatum* (0.0126), *Ligusticum Wallichii* (0.0137), *Bark of Sichuan Cork Tree* (0.0148), *Scutellaria Baicalensis* (0.0148), Dan-Shen Root (0.0151), Leaves of *P*.* notoginseng* (0.0153), Sanchi Flower (0.0153), *selfheal* (0.0155), Ginseng Flower (0.0161), *Ginseng Rhizome* (0.0161), *Ginseng Leaf* (0.0161), *Pseudo-ginseng* (0.0162), Ginseng (0.0163), *Cordyceps Sinensis* (0.0173), *Cordate Houttuynia* (0.0173), *Humulus lupulus Linn* (0.0178), *Perilla Frutescens* (0.018), Perilla Root (0.018), *Common Perilla Stem* (0.018), *Paeonia Veitchii Lynch* (0.0185), *Evodia Rutaecarpa* (0.0191), *Myrrh* (0.0198), *Paris Polyphylla* (0.0206), *Gynostemma Pentaphyllum* (0.0206), *Sarcandra Glabra* (0.0224), *Soapberry* (0.022), *Ligusticum Sinense* (0.0237), *Chinaroot Greenbrier* (0.0238), *Moutan Bark* (0.0245), *Rhizoma Ligustici* (0.0252), *Momordica Cochinchinensis* (0.0277), *Polygonatum Sibiricum Red* (0.0277), *Tripterygium Wilfordii* (0.0282), *Grass-leaved Sweetflag* (0.0284), *Willow Herb* (0.0301), *Macleaya Cordata* (0.0312), *Astragalus Membranaceus* (0.0321), *Wormwoodlike Motherwort Flower* (0.0331), *Motherwort Fruit* (0.0338), *Morinda Officinalis* (0.0338), *Motherwort* (0.0341), *Garcinia* (0.0362), *Sanguisorba Officinalis* (0.0364), *Narrow-leaved Oleaster leaf* (0.0372), *Narrow-leaved Oleaster* (0.0372), *Venenum Bufonis* (0.0384), *Radix Scrophulariae* (0.0386), *Cortex Ailanthi* (0.0403), *Adenostemma* (0.0403), *Rheum Officinale* (0.0416), *Eaglewood* (0.0435), *Trichosanthes Kirilowii Maxim* (0.044), *Pericarpium Trichosanthis* (0.044), Se*men Trichosanthis* (0.0442), *Hawthorn* (0.0454), *Cortex Acanthopanacis* (0.0457), *Radix Achyranthis Bidentatae* (0.0457), *Hawthorn* (0.0459), *Corydalis Tuber* (0.0464), *Semen Coat Lablab Album* (0.0489), *Snakegourd Root* (0.0489), *Dolichos lablab L* (0.0492), *White Hyacinth Bean* (0.0492), *Blum Blossom* (0.0492)
nfya	*Psammosilene tunicoides* (0.000338), *Concha Meretricis seu Cyclinae* (0.00182), Paeonia lactiflora (0.00728), glycyrrhiza uralensis (0.0105)
gtf2h1	Pistacia lentiscus (0.000836), honey (0.0323)
crk	*Citron* (0.00338), *Polygala tenuifolia* (0.00338), *Citrusmedica* (0.00343), *Cucumis melo* (0.00939), *Semen Melo* (0.00939), *Calyx Cucumis* (0.00941), *Manihot esculenta* (0.0129), *Ricinus communis* (0.0256)
acer3	Mimosa pudica (0.00151), Sinapis alba (0.00404)
slc4a2	Euodia rutaecarpa (0.00553), Trionycis Carapax (0.00720), Tribulus terrestris (0.00743), Coptis chinensis (0.0149)
rhoa	*Citron* (0.000211), *fructus citri* (0.000215), Rhododendron Simsii (0.00234), Indian Azalea Root (0.00234), Poria Cocos (0.0136), Radix Stephaniae Tetrandrae (0.0143), Rhynchophylla (0.0154), Pearl (0.016), Cassia Twig (0.018), Java Brucea (0.018), Astragalus Mongholicus (0.0193), Radix Rehmanniae Recen (0.0209), Cohosh (0.0226), Glutinous Rehmannia (0.0228), Astragalus Membranaceus (0.0244), White Atractylodes Rhizome (0.0259), Pistacia Lentiscus (0.0278), Dan-Shen Root (0.0311), Bird’s-nest (0.0366), *Fushen* (0.0347), Cassiabarktree Fruit (0.0351), Leaves of *P. notoginseng* (0.0356), Sanchi Flower (0.0356), Pseudo-ginseng (0.038), Oriental Wormwood (0.0387), Saposhnikovia Divaricata (0.0394), Angelica Sinensis (0.0411), Rhizoma Gastrodiae (0.0428), Ligusticum Wallichii (0.0452), Curcuma Wenyujin (0.0493)

## DISCUSSION

In this study, all corresponding public RNA sequencing data in the human postmortem prefrontal cortex were collected, merged, and reanalyzed, and 549 DEGs (147 upregulated DEGs and 402 downregulated DEGs) were identified in MDD. Subsequently, the STRING database was used to form the PPI network of DEGs, and 20 hub genes were confirmed. Furthermore, 10 of these hub genes exhibited consistent alterations in the peripheral blood of MDD patients, and 5 of these hub genes exhibited consistent changes in the specific brain regions of CUMS-treated mice showing depressive-like behaviors. We also predicted the circRNA-related posttranscriptional regulatory mechanisms of the screened hub genes and the potential anti-MDD TCMs targeting these hub genes. These findings provide some new insights into the etiology of MDD and some new potential molecular targets for its diagnosis and treatment.

MDD is the outcome of related factor abnormalities, including genetic-environmental, neuroendocrine, and gut microbiota factors, which ultimately cause some pathological changes in molecular networks and neural structures in related brain regions (Gałecki et al., 2015). Therefore, a full understanding of the molecular substrates of MDD pathogenesis, especially in the brain, is more important for MDD diagnosis and treatment. This work first combined 2 datasets to obtain a relatively larger number of human brain samples from MDD patients and controls, and 549 DEGs were then identified in the prefrontal cortex of MDD patients compared with that of controls. GO analysis revealed that these DEGs primarily regulate dendritic spine formation and synaptic architecture, a function closely related to MDD (Duman et al., 2016). KEGG analysis showed that the DEGs were mainly enriched in the linoleic acid metabolism, sphingolipid signaling pathway, and protein processing in the endoplasmic reticulum. Previous studies have shown a substantial correlation between linoleic acid metabolism and the etiology/therapy of MDD (Pu et al., 2021). Similarly, studies have shown that sphingolipids are abundant in the central nervous system and that their metabolites play a role in controlling cell division and apoptosis. Downstream signaling pathways have a significant impact on the prevalence and progression of MDD (Kitatani et al., 2008; Bienias et al., 2016). Increasing evidence shows that dysregulated protein processing in the endoplasmic reticulum, which results in the buildup of misfolded proteins and causes endoplasmic reticulum stress, plays a significant role in the pathophysiology of depression (Gold et al., 2013). The results from the KEGG pathway and GO enrichment analyses also suggest that the screening strategy used in this study is feasible and effective and that the results are reliable.

In addition, we used human peripheral blood from MDD patients to evaluate whether any of the screened hub genes might serve as diagnostic biomarkers for MDD. In MDD patients, the expression of the *KDM6B*, *CUX2*, *NAAA*, and *PHKB* genes was considerably higher, whereas that of the *NFYA*, *GTF2H1*, *CRK*, *CCNG2*, *ACER3*, and *SLC4A2* genes was markedly reduced. These findings are consistent with the expression alterations in brain tissue, suggesting their usefulness in the future diagnosis of MDD. However, receiver operator characteristic curve analysis of these genes should be performed with a larger population in the future.

Furthermore, the expression of the *Kdm6b*, *Arid1b*, *Scaf11*, and *Thoc2* genes was significantly increased in the prefrontal cortex of CUMS-treated mice, whereas the expression of the *Ccng2* gene was significantly decreased. These alterations in gene expression are also consistent with the changes observed in the cerebral cortex of humans. *KDM6B* and *ARID1B* have been demonstrated to be important in the regulation of depression. *KDM6B* regulates macrophage involvement in the inflammatory response while influencing cell differentiation and development (Qin et al., 2021). *ARID1B* is a component of the Brg/Brm-related factor (BAF) chromatin remodeling complex in the brain, and Arid1b haploid sufficiency interferes with neuronal development and leads to cognitive and emotional impairment (Smith et al., 2020). Studies have suggested that *SCAF11* may be associated with programmed cell death caused by inflammatory vesicles (Ning et al., 2022). *THOC2* encodes the *THOC2* protein, whose subunit variants can lead to neurodevelopmental disorders by interfering with mRNA export from the nucleus to the cytoplasm (Kumar et al., 2015). The cell cycle protein *CCNG2* gene encodes G2, aberrant G2/M phase control may play a degenerative role in the death of dopamine neurons, and misregulation may be associated with neuronal apoptosis (Bajić et al., 2011). Studies have revealed that patients with depression exhibit hippocampal damage, which is associated with increased apoptosis of hippocampal neurons (Gulyaeva, 2019). Therefore, it is highly possible that the *CCNG2* gene is involved in the onset and development of depression. Although these studies suggest that the aforementioned genes may be closely associated with the development of depression, their exact mechanisms need to be further investigated. Our findings imply that *KDM6B*, *ARID1B*, *SCAF11*, *THOC2*, and *CCNG2* are suitable genes for further investigation of the pathophysiology and diagnosis of MDD.

We also predicted the posttranscriptional regulatory mechanisms responsible for the changes in the expression of hub genes. CircRNAs, a type of covalently closed endogenous single-stranded RNA, may bind to miRNAs in a competitive manner and function as sponges to impede the interaction between miRNAs and their target genes (Salmena et al., 2011; Karreth and Pandolfi, 2013; Thomson and Dinger, 2016). In this study, the LncRNADisease database was first used to identify 5 circRNAs with a strong correlation with MDD, and their interacting miRNAs and downstream target genes were then predicted. The intersection of these target genes with the 20 hub genes thus allowed the creation of a circRNA-miRNA-mRNA regulatory network, which may be helpful for clarifying the regulatory mechanisms upstream of these hub genes in the future. Certainly, many experiments are still needed to verify whether these molecules can actually interact with each other and to elucidate their detailed mode of action.

According to data from an evidence-based medicine perspective, TCM has achieved significant effects on MDD treatment (Shao et al., 2020). Therefore, the identification of novel herbs with antidepressant effects and the development of new prescriptions for clinical MDD treatment are valuable. An analysis of the Coremine Medical platform based on the screened hub pathogenic genes identified some potential anti-MDD TCMs, such as citron, fructus citri, *Panax notoginseng* leaves, sanchi flower, pseudoginseng, and dan-shen root. Further analysis showed that the antidepressant effects of TCMs might be closely related to their abilities to modulate inflammation. Sawamoto et al. indicated that citron treatment could attenuate the corticosterone-induced decrease in anti-doublecortin expression in the hippocampus, which might contribute to incremental improvements in neuronal information processing and emotional recovery (Sawamoto et al., 2016). Fructus citri also has immunomodulatory effects, and through these effects, its aqueous extract enhances the functions of the hypothalamus-pituitary-thyroid and hypothalamus-pituitary-adrenal axes of rats with depressive-like behaviors to varying degrees (Zhang et al., 2007). Saponins are one of the most important constituents in the leaves of *P. notoginseng*, sanchi flower, and pseudoginseng. Many investigations have shown that the total saponins and other components of *P. notoginseng* exert a neuroprotective effect through anti-inflammatory or antioxidant activities (Xiang et al., 2011; Su et al., 2014; Zhang et al., 2016). The primary active element of dan-shen root is salvianolic acid B, which has powerful antioxidant and free radical–scavenging properties and may be utilized to prevent and cure neurological disorders in addition to cardiovascular and cerebrovascular ailments (Gu et al., 2018).

However, it should be noted that this study has some limitations. First, although we combined data from 2 public datasets to greatly increase the number of MDD brain tissue samples and restricted the range of brain regions to the prefrontal cortex, the sample size in this study was still small due to the existing limitations of collecting human brain samples for MDD study. Moreover, the differences in some factors, such as sex, age, and race, in these 2 datasets may also interfere with the final result, and the conclusions should be interpreted with caution. Finally, in addition to rigorous bioinformatics analysis, despite additional data from peripheral blood samples, a self-constructed CUMS-treated mouse model and an independent drug screening platform were used to evaluate the pathogenicity, diagnostic efficacy, and ability to predict drugs for the screened hub genes. Our research is a preliminary study, and more in vitro and in vivo experiments are needed for further verification. Fortunately, our work may greatly define the candidates and improve the success rate of future experiments.

## CONCLUSION

In summary, through bioinformatics analysis of brain RNA-seq data from MDD patients, new susceptibility genes for MDD were identified. Among these genes, we also found some molecules that are expected to be used in future studies of body fluid–based diagnosis and pathogenesis of MDD. This study may not only deepen our understanding of the etiology of depression but may also shed light on its diagnosis and treatment.

## Data Availability

The data underlying this article will be provided on reasonable request to the corresponding author.
